# Validation of a Coarse-Grained Martini 3 Model for Molecular Oxygen

**DOI:** 10.1021/acs.jctc.4c01348

**Published:** 2024-12-30

**Authors:** Samaneh Davoudi, Petteri A. Vainikka, Siewert J. Marrink, An Ghysels

**Affiliations:** † IBiTech - BioMMedA Group, Ghent University, Corneel Heymanslaan 10, Entrance 98, 9000 Gent, Belgium; ‡ Centre for Analysis and Synthesis, Lund University, Naturvetarvägen 22/Sölvegatan 39 A, 223 62 Lund, Sweden; § Molecular Dynamics Group, Groningen University, Nijenborgh 7, 9747 AG Groningen, The Netherlands; ∥ IBiTech − BioMMedA Group, Ghent University, Corneel Heymanslaan 10, Entrance 98, 9000 Gent, Belgium

## Abstract

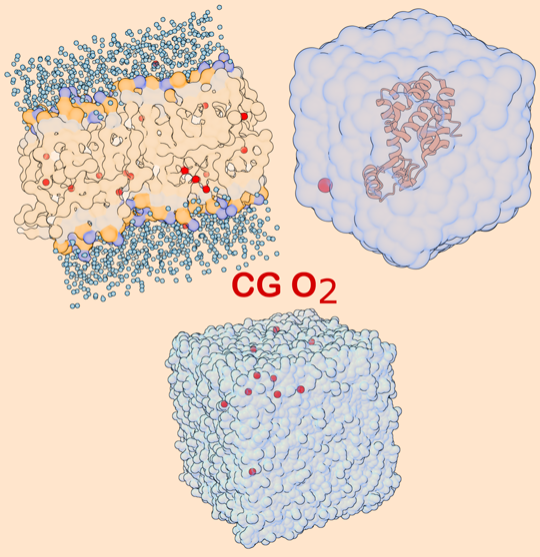

Molecular oxygen (O_2_) is essential for life, and continuous effort has been made to understand its pathways in cellular respiration with all-atom (AA) molecular dynamics (MD) simulations of, e.g., membrane permeation or binding to proteins. To reach larger length scales with models, such as curved membranes in mitochondria or caveolae, coarse-grained (CG) simulations could be used at much lower computational cost than AA simulations. Yet a CG model for O_2_ is lacking. In this work, a CG model for O_2_ is therefore carefully selected from the Martini 3 force field based on criteria including size, zero charge, nonpolarity, solubility in nonpolar organic solvents, and partitioning in a phospholipid membrane. This chosen CG model for O_2_ (TC3 bead) is then further evaluated through the calculation of its diffusion constant in water and hexadecane, its permeability rate across pure phospholipid- and cholesterol-containing membranes, and its binding to the T4 lysozyme L99A protein. Our CG model shows semiquantitative agreement between CG diffusivity and permeation rates with the corresponding AA values and available experimental data. Additionally, it captures the binding to hydrophobic cavities of the protein, aligning well with the AA simulation of the same system. Thus, the results show that our O_2_ model approximates the behavior observed in the AA simulations. The CG O_2_ model is compatible with the widely used multifunctional Martini 3 force field for biological simulations, which will allow for the simulation of large biomolecular systems involved in O_2_’s transport in the body.

## Introduction

Measuring oxygen (O_2_) distributions across the cell remains a major challenge for experiments, where the spatial resolution is not on par with the subnanometer size of the O_2_ molecule. Fortunately, *in silico* molecular dynamic (MD) simulations at the atomic scale can provide both spatial and temporal resolution.^1−10^ For simulations to be meaningful, the underlying interaction model, also called the force field, needs to describe the intermolecular interactions sufficiently accurately. In theory, high accuracy could be achieved with quantum mechanical calculations, but in practice, such calculations are computationally too expensive.

Instead, much cheaper classical all-atom (AA) models have been successfully used to investigate interaction of O_2_ with membranes and proteins. Marrink et al. employed the AA GROMOS force field with slight modifications to calculate O_2_ permeability through phospholipid membranes, the O_2_ diffusion constant in solvents, and the O_2_ solubility in solvents.^11^ Also, the AA CHARMM36 force field^12^ for lipid molecules and proteins was extended with an O_2_ model,^13^ and the diffusion of O_2_ through a lipid membrane was investigated in various studies. These AA MD simulations gave insight into the position-dependent anisotropy of O_2_’s diffusion pathways, parallel versus orthogonal to the membrane,^13^ as well as the characteristic time and length scales of membrane permeation.^14^ Other investigated aspects were the impact of lipid chain unsaturation,^15^ temperature,^14^ lipid type,^16^ lipid phase,^9^ and membrane stacking^17^ on O_2_ diffusion through the lipid bilayers. More recently, to shed light on the effect of caveolae’s role on O_2_ buffering, Davoudi et al. have explored the effect of membrane curvature on O_2_ partitioning through lipid membranes.^18,19^ The AA CHARMM force field also has several slightly different AA interaction models. In a comprehensive study, Javanainen et al. compared 14 AA molecular oxygen models that were available at that time, including several other AA CHARMM models.^20,21^ They also developed several new oxygen models, with and without quadrupole moment, which, however, still have some difficulties in reproducing the experimental free energy of transfer to hexadecane, a solvent that imitates the interior of phospholipid bilayers. This property thus remains a target for further AA force field developments.

The AA Amber force field^22^ has also been extended for oxygen simulations with two oxygen versions, i.e., a general Amber force field (GAFF) version and an updated version with adapted Lennard-Jones parameters.^23^ Pias and co-workers gave important contributions to map out the impact of cholesterol (CHOL) on O_2_ permeability,^23−26^ and they analyzed the pathway by which O_2_ travels through cells and tissues.^27,28^ Furthermore, their investigations have extended to the examination of how incorporating a sodium channel into the membrane affects O_2_ permeability.^29^ Questions remain, however, about how oxygen interacts with curved structures, such as caveolae, which are membranes populated by curvature-inducing transmembrane proteins,^30^ entire membrane structures such as mitochondria,^31^ or extended myelin sheaths in the nervous system.^17,32^

The interaction of O_2_ with proteins has also been investigated by various research groups. In the study by Polyakov et al., the interaction of O_2_ with the flavin-dependent protein miniSOG was investigated, revealing the formation of singlet oxygen and superoxide upon light illumination.^33^ Through AA MD simulations and quantum mechanics/molecular mechanics (QM/MM) approaches, they identify several O_2_-binding pockets, enhancing the understanding of miniSOG’s photophysical properties as a key singlet O_2_ photosensitizer. In another study, using Car–Parrinello MD simulations, it is revealed that O_2_ significantly weakens the binding strength of carbon monoxide (CO) to hemoglobin. Their findings offer new therapeutic insights for CO poisoning.^34^ Lepeshkevich et al. also investigated O_2_ migration in isolated β-chains of human hemoglobin using MD simulations and laser kinetic absorption spectroscopy. They found that the insertion of xenon (Xe) atoms decreases the time constant of O_2_ rebinding due to reduced intraprotein space, providing insights into the binding mechanisms of both native hemoglobin and artificial O_2_ carriers.^35^

However, when dealing with larger simulations comprising millions of atoms, the computational burden and time required for AA simulations can become prohibitive. To address this challenge, employing lower-resolution force fields such as the coarse-grained (CG) Martini model offers a practical solution.^36^ In the Martini model, atoms are grouped together into beads, thereby reducing the computational load. The Martini model is a widely used CG general-purpose force field, employed in a broad range of applications, including membrane permeation and binding of small molecules to proteins.^37−40^ The current version of Martini is denoted Martini 3.^41^ Many validated Martini 3 topologies are available for small molecules in general,^42^ but for gases only a model for nitrogen is available compatible with the previous version, Martini 2.^43^

A CG model of O_2_ is still lacking in Martini 3. Therefore, in this work, we aim to develop a Martini 3 O_2_ model, where the two atoms of molecular O_2_ are lumped together into a single bead. The interactions of this bead with lipid molecules and water are responsible for membrane–water partitioning and potential buffering of the O_2_ in the membrane. Moreover, this bead’s interaction with amino acids should lead to a binding preference for hydrophobic cavities of proteins instead of hydrophilic ones. The proposed CG bead with its interactions will therefore be evaluated by testing a set of criteria, such as the bead’s partitioning in oil–water and in membranes, its diffusivity in solvents, and the vacancy occupancy in the T4 lysozyme L99A protein.

In terms of the long-term impact, the CG Martini model for O_2_ will facilitate diverse simulations of biosystems and O_2_ at the CG level, significantly reducing computational expenses for larger systems. It will enable us to go beyond the modeling of flat membranes,^44^ and the simulation box may even encompass curved membranes and liposomes.^45^ A potential application is to gain insight into the dynamics of O_2_ buffering in caveolae, which are highly curved invaginations in the heart cell membranes, requiring a large simulation box to capture the complex membrane structure. Another application could be to explore high-throughput identification of potential oxygen-binding pockets across a large database of proteins.

## Methods: Selection from Existing CG Beads

### Size, Charge, Polarity, and Oil–Water Partitioning

An oxygen molecule has a diameter of approximately 3.4 Å and a molar mass of 32 g/mol.^46^ The parametrization of a new CG model for O_2_ would entail establishing interactions between the new CG representation and existing beads and tedious validation. Therefore, it may be more efficient to identify analogous beads among those already established and validated, thereby avoiding a new parametrization process. To identify a bead analogous to O_2_ among existing Martini 3 beads, our attention was directed toward the smaller CG beads, particularly those of tiny size (label ‘T’) with σ (the distance at which the intermolecular potential between the two particles is zero) equal to 3.4 Å and a standard assigned molar mass of 36 g/mol. Molecular O_2_ is a noncharged and nonpolar molecule, with its lowest moment being the quadrupolar moment. Within the subset of tiny beads, two bead types meet the conditions of being noncharged and nonpolar: the TC and TX bead types are two promising candidates to represent O_2_. The Martini 3 force field distinguishes six TC beads and four TX beads, each characterized by a polarity index ranging from 1 (minimal polarity) to 6 (maximal polarity) for TC beads and similarly from 1 to 4 for TX beads.

Next, the group of these ten Martini 3 beads will be further narrowed down based on a comparison of the O_2_ solubility in water versus a nonpolar solvent. As noted by Overton more than a century ago,^47,48^ the membrane–water partitioning correlates well with oil–water partitioning, such as octane–water (OCO-W) or hexadecane–water (HD-W) partitioning. Therefore, Table 1 compares a set of partitioning values of the Martini 3 beads, values from AA simulations of O_2_, and experimental values. The partitioning is quantified by the free energy Δ*G*_transfer_ required for the oxygen molecule to transfer from one phase to another.

**Table 1:** Comparison of the Selected CG Beads with the O_2_ Molecule in AA MD and Experiments: O_2_ Gas Density and Δ*G*_transfer_ for Water–Solvent Partitioning^a^

		Δ*G*_transfer_ (kJ/mol)				
	density (kg/m^3^)	HD-W	OCO-W	ETH-W	BENZ-W	CHEX-W
Martini 3 (TC1)^41^		13.2	12.0	10.5	8.2	13.2
Martini 3 (TC2)^41^		11.1	7.8	7.9	4.8	9.6
Martini 3 (TC3)^41^		8.6	6.7	8.2	5.5	8.5
Martini 3 (TC4)^41^		5.6	6.4	8.1	8.0	8.5
Martini 3 (TC5)^41^		3.6	4.5	6.4	6.3	5.7
Martini 3 (TC6)^41^		1.3	3.6	6.4	5.0	4.3
Martini 3 (TX1)^41^		8.4	7.6	9.3	9.1	9.6
Martini 3 (TX2)^41^		5.3	5.2	6.4	3.7	5.5
Martini 3 (TX3)^41^		5.4	5.4	7.5	3.6	5.5
Martini 3 (TX4)^41^		1.2	2.7	7.4	2.5	4.3
Martini 3 (TC3)^310 K^	1.25 ± 0.01^b^					
AA CHARMM (O_2_)^13^^,310 K^		7.5				
AA CHARMM^c^ (O_2_)^21^^,298 K^		6.4–8.3 ± 0.04–0.07				
exp. (O_2_)^49^	1.30^50^	10.8	9.7	8.0	8.9	9.9
exp. (O_2_)^49^^,310 K^		11.0	10.5	8.8	9.7	10.8
exp. (O_2_)^51^^,273.15 K^	1.42					
exp. (O_2_)^52^				8.08 ± 0.11	7.63 ± 0.10	7.21 ± 0.03
exp. (O_2_)^53^^,295 K^		5.2				

a Error bars for the Martini-based free energies are 0.2 kJ/mol. Error bars for the simulation AA and experimental values are given where available. All values are at 300 K (simulation values at 1 bar), unless specified otherwise.

b Indicates the value is calculated from new simulations by our group at 310 K.

c Indicates the range of values from different versions of the CHARMM force field.

In Table 1, the coarse-grained (Martini 3) partitioning values are taken from the Supporting Information of the 2021 reference paper.^41^ An additional TC3 gas density calculation with a Martini 3 was performed at 310 K for this work. Various solvents were considered in the Martini 3 paper: hexadecane (HD), octanol (OCO), ether (ETH), benzene (BENZ), and cyclohexane (CHEX). The all-atom value (AA CHARMM) for hexadecane–water partitioning was taken from the O_2_ study by Ghysels et al.,^13^ and the label “AA CHARMM” will refer to this specific CHARMM oxygen model in the remainder of the paper. The table also includes the range of transfer-free energies Δ*G*_transfer_ for different AA CHARMM versions, as reported in detail by Javanainen et al.^20,21^

Let us introduce the experimental values in Table 1. The free energy values for transferring (Δ*G*_transfer_) a permeant from a nonpolar solvent (*S*) to the water (*W*) phase were calculated based on the difference between a hydration and solvation energy, which both have the gas (*G*) phase as a starting point^54^
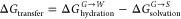
1Here, Δ*G*_hydration_^*G* → *W*^ is the free energy associated with the transition of a permeant from the gas phase to a water environment, while Δ*G*_solvation_^*G* → *S*^ is the free energy change as the permeant shifts from the gas phase to the solvent phase. The two free energy values Δ*G*_hydration_^*G* → *W*^ and Δ*G*_solvation_^*G* → *S*^ are not directly tabulated for experiments. Instead, they were computed from the relation with the solubility
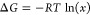
2where *x* represents the mole fraction solubility in the water or the various solvents. The mole fraction solubility values were sourced from the literature at a partial pressure of 101.325 kPa for the gas and at 300 K.^49^ Similarly to the Martini 3 paper,^41^ various solvents were considered. Following the calculation of Δ*G*_hydration_^*G* → *W*^ and Δ*G*_solvation_^*G* → *S*^ with eq 2, the resulting Δ*G*_transfer_ values (eq 1) are presented in Table 1.

Together, the values in Table 1 give an overview of the interactions of O_2_ with the water phase and the solvents. Specifically for oxygen interacting with a membrane, the HD partitioning is most relevant as it contains the same number of carbon atoms (16 carbons) in the lipid tail as 1-palmitoyl-2-oleoyl-*sn*-glycero-3-phosphocholine (POPC).^13,25^ The AA CHARMM simulations give a value of Δ*G*_transfer_ = 7.5 kJ/mol for HD-W partitioning. From the ten TC and TX Martini 3 beads, two beads (TC3 and TX1) exhibit the closest resemblance in HD partitioning free energy (8.6 and 8.4 kJ/mol, respectively) compared to the HD partitioning free energy for AA CHARMM O_2_. Similarly, TC2 shows good agreement with experimental data with an HD partitioning free energy of 11.1 kJ/mol. These three beads (TC2, TC3, and TX1) are thus good candidates as a CG model for O_2_ based on their size, zero charge, nonpolar character, and hexadecane–water partitioning. At the same time, these three bead types also reproduce the partitioning free energy into other organic solvents reasonably well in comparison with available experimental data (Table 1).

### Solubility of O_2_ in the Membrane

In this subsection, it will be investigated how the three selected beads (TC2, TC3 and TX1) perform in reproducing the partitioning of the O_2_ in a POPC membrane. Given our objective to model oxygen’s interactions with membranes, the final selection among the remaining beads will be determined by evaluating their solubility in a membrane against their solubility in the water phase. This ensures a sufficiently accurate representation of the water–membrane interactions of the modeled oxygen.

The simulated flat membrane composition is a homogeneous POPC bilayer, as phosphocholine is the most abundant phospholipid in all mammalian cell membranes.^55^ As a second membrane composition, a molar fraction of 25% cholesterol was considered.

**Figure 1:**
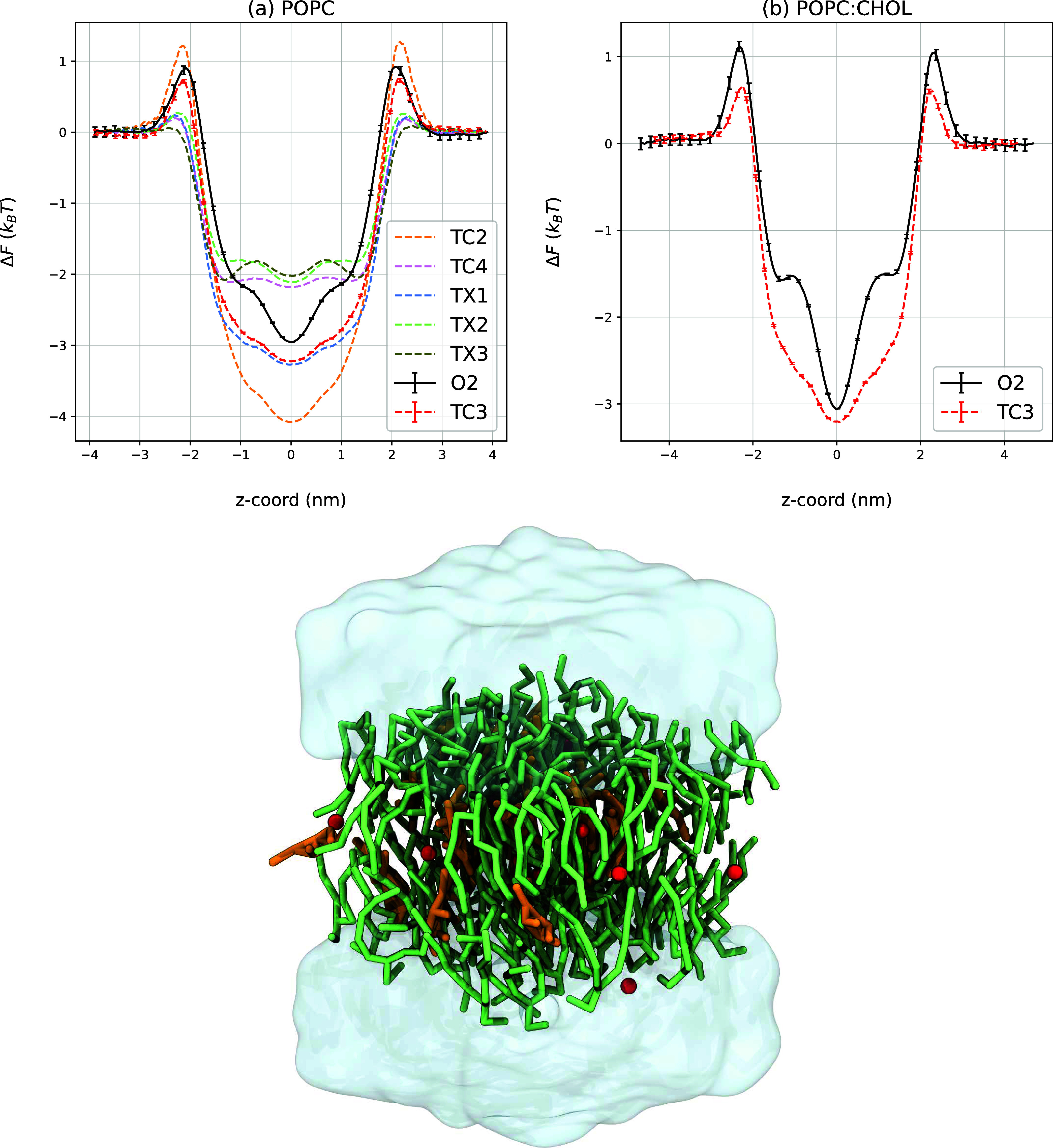
Top panel: free energy *F*(*z*) of oxygen partitioning through (a) POPC and (b) POPC:CHOL flat bilayers as a function of oxygen’s position *z* along the membrane normal. The center of the bilayer is located at *z* = 0. Profiles are shifted to *F* = 0 in the water phase. (a) Comparison for a few Martini 3 beads (dashed line) and AA CHARMM O_2_ (black solid line) in POPC. (b) Comparison for selected TC3 beads (red) and AA CHARMM O_2_ (black) in POPC:CHOL. Errors estimated from block averaging with 10 blocks are shown for AA and TC3. Bottom panel: Flat membrane model featuring POPC (green), cholesterol (yellow), Martini 3 wt % O_2_ (red), and water (light blue, surface).

The free energy associated with the transfer of O_2_ from a water environment to a POPC or POPC:CHOL bilayer was calculated. Identical systems were built with either an AA force field (AA CHARMM) or a CG (Martini 3) representation of O_2_ (see the Computational Details section). The solubility can be visualized by the free energy profile along the membrane normal, i.e., *F*(*z*) = −*k*_B_*T *ln(*hist*(*z*)), where *hist*(*z*) refers to the positional histogram of oxygen’s center of mass position *z* along the membrane normal. The free energy profile *F*(*z*) was computed as in ref (56). and is given in Figure 1. The free energy difference Δ*G*_*m*_ to transfer from the center of the membrane (*z* = 0) to the water phase is included in Table 5. To confirm the suitability of our selected beads, we included some of the other CG beads in this part and compared them to O_2_ as well.

The TX1, TX2, and TX3 beads lack the barrier of about 1 *k*_*B*_*T* to enter the POPC membrane, located around |*z* |= 2 nm, which would lead to an overestimation of the entrance kinetics of oxygen into the membrane (Figure 1a). Among the TC2, TC3, and TC4 beads, beads TC2 and TC3 exhibit a reasonable entrance barrier (Figure 1a). The TC3 bead, however, has a much better agreement with the AA free energy well than TC2, whose well is too deep. This free energy well is important for partitioning in the membrane and the residence time and escape kinetics of oxygen from the membrane. In conclusion, the preselected TC3 bead is indeed a very reasonable choice to mimic the membrane–water partitioning of O_2_. The match is not perfect, however. In the CG simulation, the free energy plot displayed a deeper well (0.31 *k*_*B*_*T* deeper) and a smaller barrier (0.19 *k*_*B*_*T* smaller) compared to the AA simulation, indicating that somewhat higher partitioning in the membrane, slower escape kinetics, and faster entrance kinetics can be expected for TC3 compared to AA simulations.

Similarly, the free energy Δ*G*_*m*_ was also modeled for transferring TC3 from water to a heterogeneous POPC:CHOL bilayer with 25% cholesterol. The free energy profile across the membrane normal is compared to that of the corresponding AA system with the same composition in Figure 1b. The free energy barrier in the headgroup region around |*z* | = 2.1 nm for the CG differs from AA simulation (0.52 *k*_*B*_*T* smaller). The small shoulders in the tail region of the AA free energy (|*z* | ≈ 1 nm) are absent in the CG free energy, but the profiles show a good match in the depth of the free energy at *z* = 0.

In summary, the membrane–oxygen interactions led to the decision to select TC3 as the bead to represent molecular O_2_. For completeness, we also computed the molecular oxygen gas density for TC3 at 310 K, which matches well with the experiment (see Table 1). In the next section, the TC3 bead will be evaluated for its other properties, such as hydration-free energy of other solvents, oxygen diffusivity in a homogeneous phase, oxygen permeability through a membrane, and oxygen–protein interactions.

## Further Comparison of AA and CG

### O_2_ Diffusivity in Oil and Water

The O_2_ diffusivity in water and hexadecane is compared between CG, AA, and experiment in Table 2. The CG simulation uses the TC3 bead in a cubic water box, while the AA simulation uses the AA CHARMM force field (see the Computational Details section). The diffusivities were corrected for the periodic boundary effects (see Section 5.2.3). The CG diffusivity of O_2_ in water is 0.731 Å^2^/ps, which aligns well with the AA CHARMM simulation conducted by Ghysels et al.^13^ However, both CG and AA diffusivities deviate from the experimental values (0.19 to 0.252 Å^2^/ps).^53,57,58^ The same trend is observed for O_2_ diffusivity in hexadecane, with the CG (0.575 Å^2^/ps) and AA (0.47 Å^2^/ps) values exhibiting a fair resemblance but differing from the experimental value (0.249 Å^2^/ps).^13^

**Table 2:** Comparison of Oxygen Diffusion Coefficient in Water (*W*) and Hexadecane (HD) between CG Simulations (TC3, Martini 3), AA Simulations (O_2_ AA CHARMM), and Experimental O_2_ Data^a^

systems	temperature	in *W* (Å^2^/ps)	in HD (Å^2^/ps)
Martini 3 (TC3)^b^	310 K	0.714 ± 0.06	0.566 ± 0.02
		0.731 ± 0.06^c^	0.575 ± 0.02^c^
AA CHARMM (O_2_)^13^	310 K	0.60	0.44
		0.66^c^	0.47^c^
exp. (O_2_)^53^	295 K	0.211	0.249
exp. (O_2_)^57^	308 K	0.252 ± 0.06	
exp. (O_2_)^58^	308 K	0.26–0.29	

a Simulations at 1 bar and experiments at 1 atm.

b Refers to values from new simulations by our group.

c Refers to values for CG and AA systems after correction for periodic boundary conditions.

This difference between simulations and experiments can be because simulations are usually sufficiently accurate for thermodynamic properties, but they have difficulties reproducing kinetic properties accurately. Variations in temperature and pressure, inaccuracies in the force field, and neglect of quantum effects contribute to deviations from the experimental results. It is, for instance, well-documented that the popular TIP3P model for all-atom water overestimates the water diffusivity in water by roughly a factor of two. It is therefore not surprising that the AA CHARMM model for oxygen cannot accurately reproduce experimental diffusivities.^13^

Interestingly, the Martini 3 diffusivities do succeed in reproducing the AA CHARMM diffusivity with acceptable accuracy in Table 2, despite the additional approximations implied by the coarse-graining. Moreover, the CG diffusion constants reproduce the AA trend of a higher diffusivity in water than in hexadecane. The loss of degrees of freedom in the CG model is often expected to speed up the diffusion compared to an AA simulation, in particular for polar solvents.^59^ However, because Martini water beads represent four water molecules, the effective viscosity of Martini water is close to real water. Another contribution partially negating the speedup is due to the mass of the TC3 bead (36 g/mol), which is slightly higher than molecular oxygen (32 g/mol). These competing effects ultimately give an acceptable kinetic behavior of the CG O_2_ bead in both water and hexadecane compared to the AA CHARMM O_2_.

### O_2_ Permeability across a Lipid Membrane

The permeability *P* of O_2_ through the POPC bilayer was determined with the counting method based on the same CG (TC3, Martini 3) and AA (AA CHARMM) simulations as those for the O_2_ partitioning in a membrane. In the counting method, the permeants are followed during an equilibrium simulation, and the permeability is obtained by counting the number of full membrane crossings.^14,16,29,60,61^ The dividing surfaces, used for detecting a crossing, were positioned 8 Å further than the average position of the phosphate atom (AA) or phosphate bead (CG) in order to consider the whole membrane thickness *h* (see Table 3) as done in earlier CG simulations of membranes that include fatty acids.^56^

**Table 3:** Permeability *P* of Selected Beads through POPC and POPC:CHOL (25% cholesterol) Membranes and Position |*z*|= *h*/2 of Dividing Surfaces (with *h* Being the Membrane Thickness)^a^

	POPC		POPC:CHOL	
systems	*P* (cm/s)	*h*/2 (Å)	*P* (cm/s)	*h*/2 (Å)
CG TC2	70.1 ± 10	27.3		
CG TC3	109.1 ± 9	27.3	126.0 ± 9	28.1
CG TX1	185.5 ± 12	27.3		
CG TX2	151.8 ± 7	27.3		
AA CHARMM (O_2_)	23.7 ± 2	27.4	24.3 ± 2	29.8
AA CHARMM (O_2_)^14^	25.9	25.8		
AA CHARMM (O_2_ CHARMM-D)^62^	15.4 ± 1.8	30.0		
AA Amber (O_2_-GLJ, Berendsen)^23,25^	26 ± 2	28.0	24 ± 2	30.0
AA Amber (O_2_-L14LJ, Berendsen)^23^	18.0 ± 0.7	28.0		
AA Amber (O_2_-L14LJ, Langevin)^23^	17.0 ± 1.5	27.0		
AA Amber (O_2_-L14LJ, Langevin)^63^	13 ± 1	28.0		
exp. (O_2_)^64^	157.4	∼18.0	49.7	∼21.0

a Simulation values are at 310 K and 1 bar, experimental values are at 308 K and 1 atm.

Given that TC3 exhibited the most analogous free energy profile to O_2_, we also calculate the permeability of TC3 through the POPC:CHOL membrane. Moreover, our resulting simulation values in Table 3 were juxtaposed with data from other published AA simulations. In earlier simulation work with the AA CHARMM force field, the permeability of POPC was not determined with the counting method, but based on Bayesian analysis of MD trajectories.^14^ A recent study by Shinn and Tajhkorshid imposed a concentration gradient in the simulation box with a biasing force and counted the transitions in one direction in this nonequilibrium simulation.^62^ They used the AA CHARMM-D force field, where the quadrupolar oxygen molecule is modeled with two dipoles,^2,65,66^ and varied the oxygen concentration.

Another set of permeability values in Table 3 are simulation results with the AA Amber force field for the O_2_ reported by Pias and co-workers. They used the Lipid14 force field for the bilayer,^67^ TIP3P for water,^68^ different oxygen concentrations, and two versions for the AA oxygen interactions. In the O_2_-GLJ version, the Lennard-Jones parameters originate from the General Amber Force Field (GAFF) parameters for the O atom type.^25^ In the O_2_-L14LJ version, the Lennard-Jones parameters were adjusted to match the ether carbonyl oxygen atom type (oC) in the Lipid14 force field.^23,63^ The results for both versions are shown in Table 3. The dividing surfaces for *P* calculations were set at 28 Å for POPC and 30 Å for POPC:CHOL, fairly similar to our simulations. Note however that the Berendsen or Langevin thermostat was used in these works, which affects the permeation kinetics, in contrast to the canonical sampling through a velocity rescaling thermostat (Bussi–Donadio–Parrinello thermostat).

The experimental O_2_ permeability was determined by Widomska et al. from electron paramagnetic resonance (EPR) experiments. They measured induced spin exchanges via bimolecular collisions between O_2_ molecules and nitroxide using EPR.^64^ Experimentally, the dividing surfaces are implied to be located at 18 and 21 Å from the membrane center, so significantly deeper within the membrane compared to our chosen dividing surfaces. Overall, the *P* values derived from our CG simulations of modeled O_2_ permeation through the membrane, ranging from 70 to 152 cm/s, exhibit a closer resemblance to experimental values, which fall within the range of 49 to 190 cm/s. This contrasts with the AA simulation data, which span from 10 to 26 cm/s. As previously mentioned, experimental *P* determination relies on EPR, where the accuracy of the dividing surface position may be compromised. The more narrow boundaries in experimental setups fail to capture the significant water–lipid interfacial resistances. Consequently, the resulting experimental *P* values tend to be notably higher than those from AA simulations.

The higher *P* observed in CG simulations compared to AA simulations (CHARMM or Amber) can be attributed to the observation that CG O_2_ enters the membrane more easily than AA, given the reduced free energy barrier in the headgroup region of the membrane (Figure 1), which serves as the maximum point of resistance to oxygen permeation. The inhomogeneous solubility–diffusivity model for permeation contains contributions from both energetic (*F*) and kinetic (*D*) effects. Given that the diffusivities of AA and CG are quite similar in both aqueous and oil-like solvents, it seems reasonable to focus on the (exponential) contribution of the free energy rather than the diffusivities to explain the difference in *P*.

The permeability in the CG system increases slightly upon the introduction of 25% CHOL into the membrane, whereas changes are not statistically significant in the AA systems (Table 3). When CHOL is introduced in the membrane, a small free energy shoulder appears in the AA simulation near the center of the membrane, but this shoulder is absent in the CG simulation (Figure 1b). Additionally, the introduction of CHOL reduces the free energy barrier in the headgroup. These alterations might facilitate a smoother passage of the modeled TC3 through the membrane, resulting in a moderately increased *P* in the presence of cholesterol. The experimental values in Table 3 predict a decrease in permeability upon increasing the cholesterol.^64^ There is however still an ongoing debate about what the effect of cholesterol can be,^69^ as it depends heavily on the lipid mixture, temperature, permeant, and unfortunately also on the experimental technique.

### O_2_ Binding to Hydrophobic Cavities of T4 Lysozyme L99A

**Figure 2:**
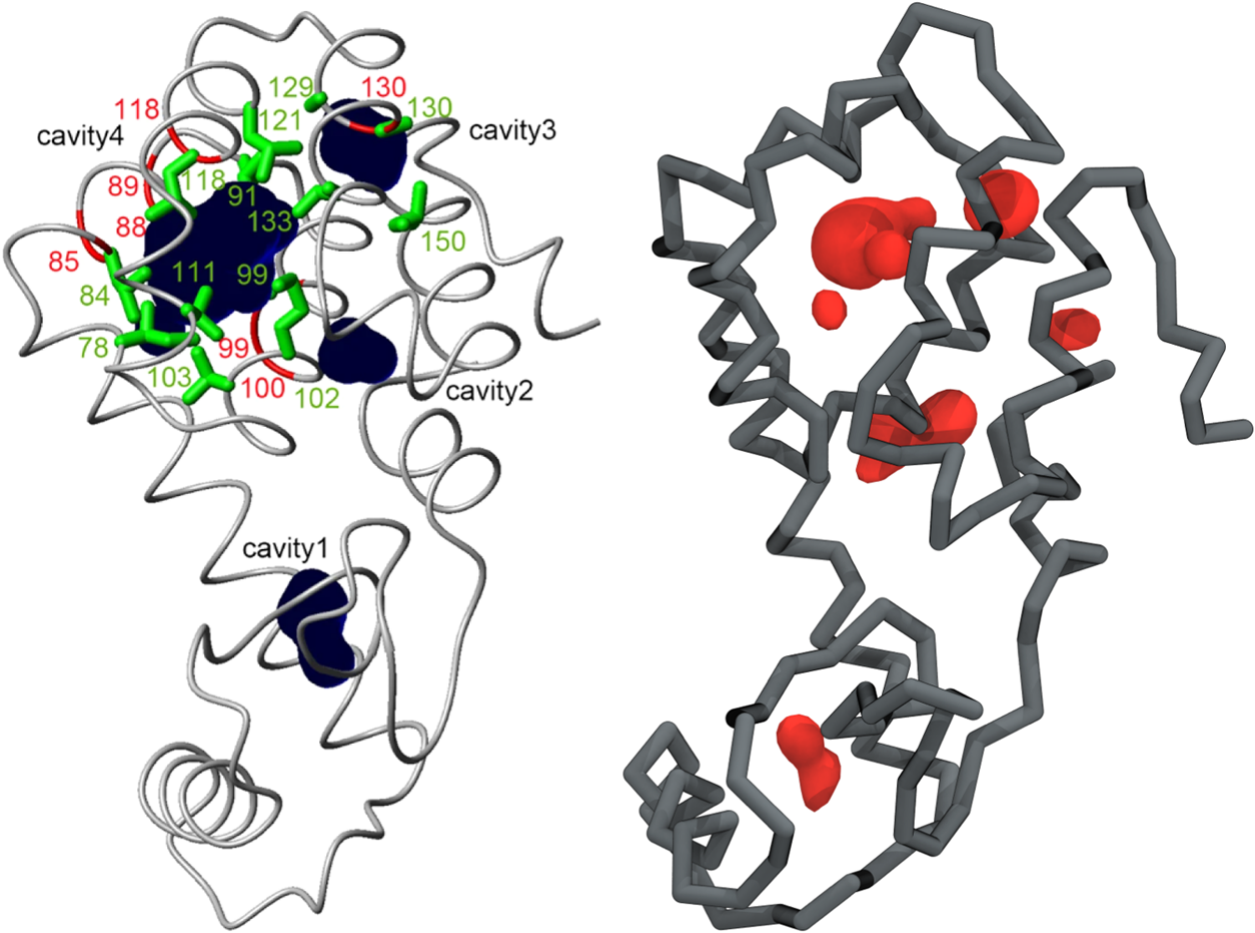
Protein T4 lysozyme L99A (gray, ribbon representation) with two hydrophilic cavities (cavity 1, cavity 2) and two hydrophobic cavities (cavity 3, cavity 4). Water and ion molecules are removed for clarity. Left panel: cavities, amide groups, and methyl groups are shown in black, red, and green, respectively. The figure is adapted with permission from ref (73), Copyright 2016 Springer Nature. Cavities calculated using the MOLMOL program.^74^ Right panel: TC3 density map (red) of the CG bead, depicted by our group.

Lysozyme, a protein abundantly found in various mucosal secretions (such as tears, saliva, and mucus) as well as in the tissues of both animals and plants, serves a crucial function in innate immunity.^70^ The T4 lysozyme L99A mutant (PDB code: 181L) stands as a prominent model system in scientific exploration, particularly in the investigation of small-molecule interactions within protein cavities.^71,72^ In the study conducted by Kitahara et al., the interaction between O_2_ and lysozyme was investigated using experimental methods and all-atom molecular dynamics, assessing the occupancy of O_2_ within two hydrophobic cavities of the protein.^73^ Their research showed that O_2_ repetitively binds to these hydrophobic cavities, denoted as cavity 3 and cavity 4, as depicted in Figure 2-left. The investigation highlights a significant difference in O_2_ binding between the two hydrophobic cavities themselves, with the binding in cavity 3 registering at only approximately 5% of that observed in cavity 4. This observation suggests a notably stronger affinity for all-atom O_2_ binding to cavity 4 compared to cavity 3. This could be attributed to the substantially larger volume of cavity 4, which is six times greater than that of cavity 3.

**Figure 3:**
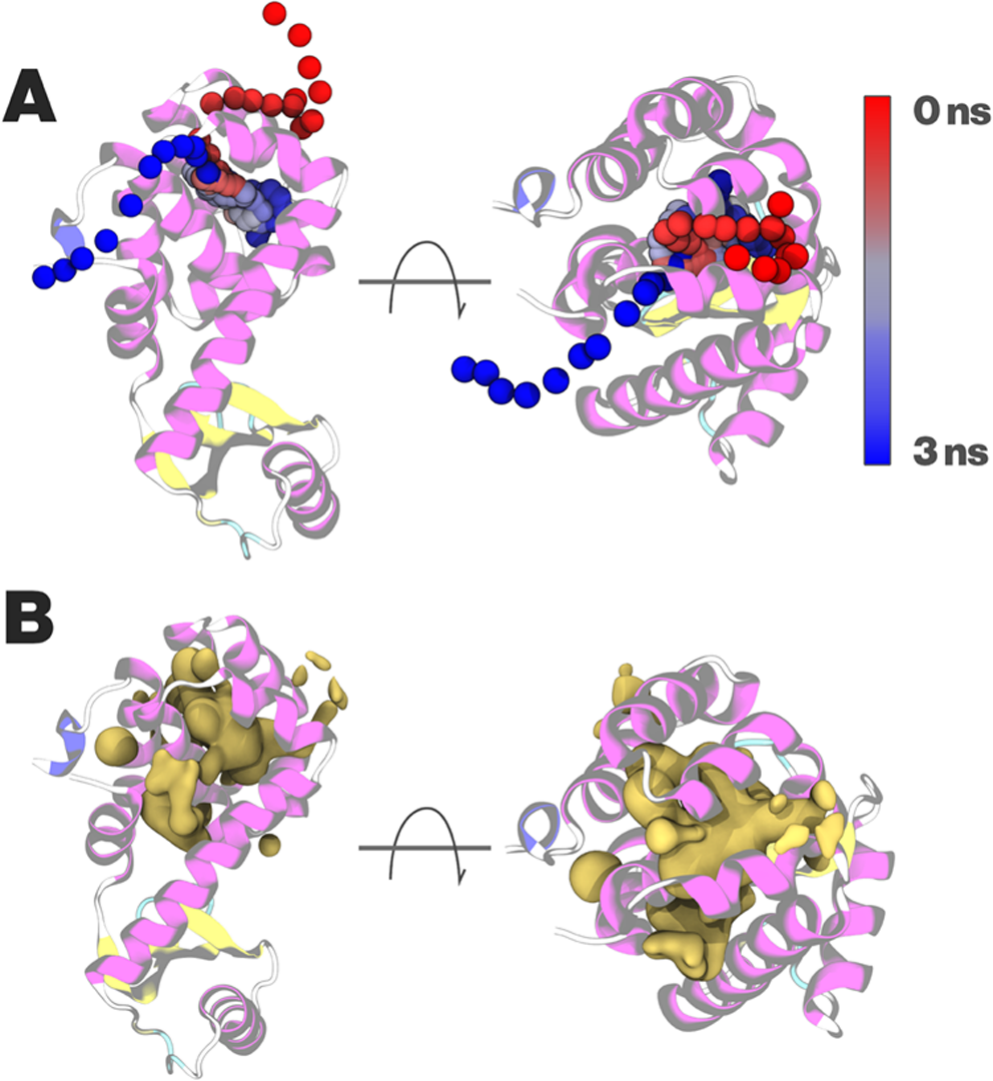
(A) A single 3 ns binding event of oxygen to lysozyme. Colorbar shows time progression. The illustrated trajectory has a 20 ps smoothing in order to reduce random noise. (B) Density isosurface of oxygen showing regions with high oxygen concentration.

To validate our CG O_2_ model (TC3 bead) against AA O_2_, we examined the binding of the modeled CG O_2_ to the hydrophilic and hydrophobic cavities of lysozyme, as well as a random space within the protein for comparison, using CG simulations (see Section 5.2.4). Our CG simulation box was identical to the AA simulation box of Maeno et al.^75^ with 2 oxygen molecules. When running 29 replicas of the simulation box of 1 μs, many binding events of O_2_ to the cavities could be observed. An example of a binding event is visualized in Figure 3. The pathway of CG O_2_ in a single 3 ns binding event with its entry in the cavity, residence, and exit from the cavity is illustrated with a colorbar (Figure 3A). The density map of O_2_ of all MD data illustrates the binding of TC3 to the protein’s cavities in Figure 2-right. Moreover, the total binding time *t*, number of binding events *n*, and average residence time *t̅* of TC3 beads in the cavities were computed from the trajectories and are presented in Table 4.

**Table 4:** Binding of Two CG O_2_ Beads to the Cavities of T4 Lysozyme L99A in 29 Replicas of 1 μs CG MD Simulation: Hydrophilic Cavity (1 & 2), Hydrophobic Cavities (3 & 4), and Random Space in the Protein (5)^a^

	cavity 1	cavity 2	cavity 3	cavity 4	cavity 5
*t̅* (ns)	0.263 ± 0.023	0.124 ± 0.010	0.542 ± 0.022	0.625 ± 0.023	0.158 ± 0.012
*n*	283	830	1098	2738	427
*t* (ns)	74.56	103.26	595.36	1710.50	67.44
occ. (%)	0.064	0.089	0.513	1.475	0.058
Δ*F*_bind_ (kJ/mol)	5.26	4.41	–0.107	–2.83	5.51

a Average residence time of occupancy by CG O_2_ beads to each cavity (*t̅*) with its standard error, total number of visits by a CG O_2_ bead to each cavity (*n*), total time of CG O_2_ beads occupying the cavity (*t*), and the percentage of occupancy of CG O_2_ beads compared to the total simulation time in each cavity (occ.) is shown. Free energy difference Δ*F*_bind_ for O_2_ between cavity and solvent.

As observed from the O_2_ density map in Figure 2-right, our CG O_2_ model predominantly occupies the hydrophobic cavities (cavity 3 and cavity 4), as should be the case for a hydrophobic oxygen molecule. The total binding time *t* to these hydrophobic cavities was about 2.3 μs in total (3836 binding events in total) in a 29 μs simulation time for both CG O_2_, as detailed in Table 4. The occupancy of our CG O_2_ model in the hydrophobic cavities is nearly 13 times higher than that in the hydrophilic cavities. These observations for TC3 are thus in agreement with the expected behavior of molecular oxygen.

While experimentally binding to the hydrophilic events is not observed, 283 binding events to cavity 1 did occur in the course of 29 μs, totaling 74.56 ns of binding time, and 830 binding events to cavity 2 occurred, totaling 177.82 ns. These unexpected binding events might be attributed to structural differences in the protein backbone between the CG and AA simulations (see gray backbone left vs right in Figure 2). Subtle changes in lysozyme’s structure can affect the hydrophobic and hydrophilic strength of the protein’s cavities. The occupancy to the random space in the protein (cavity 5) was about 9 to 25 times lower than that in the hydrophobic cavities.

Comparing the two hydrophobic cavities in Table 4, the occupancy of cavity 4 (1.475%) is nearly three times the occupancy of cavity 3 (0.513%), indicating that cavity 4 is more hydrophobic than cavity 3. The occupancy and number of binding events depend on the size of the simulation box, the number of O_2_ molecules, and the simulation time. Therefore, the free energy of oxygen-binding Δ*F*_bind_ to the different cavities was computed from the oxygen densities. This free energy is independent of the simulation choices and, thus, is a good measure of the binding affinity of the cavities. The previous observations about oxygen’s binding preference are also reflected in the free energy. The favorable binding to the hydrophobic cavities leads to a negative Δ*F*_bind_, whereas the hydrophilic and random cavities have a positive Δ*F*_bind_ due to the unfavorable affinity. The difference between the most hydrophobic cavity 4 and the most hydrophilic cavity 1 amounts to 5.85 kJ/mol. Finally, the average residence time *t̅* in the hydrophobic cavities is also significantly larger than that in the hydrophilic or random cavities, demonstrating that oxygen spends longer stretches of time in the hydrophobic cavities.

## Conclusions

To be able to investigate and simulate larger systems with the presence of O_2_ molecules, TC3 was selected among the CG Martini 3 beads to represent molecular O_2_. This selection was based on a comparative analysis of several properties.

Initially, 10 CG beads were selected based on their size, charge, and nonpolarity. Next, the free energy (Δ*G*_transfer_) of O_2_ solubility for these ten CG beads was compared with both AA CHARMM simulations and experimental values in nonpolar organic solvents. Among them, the TC2, TC3, and TX1 beads showed free energies that were closest to those of O_2_ in AA CHARMM simulations and experiments. Next, the free energy of these three beads traversing the membrane (POPC and POPC:CHOL) was graphed along the membrane normal and compared with that of AA CHARMM O_2_. The TC3 bead showed the most analogous behavior to the free energy profile of AA CHARMM O_2_. It matched the free energy barrier, which dictates the kinetics of O_2_ entering the membrane, and it matched the free energy well at the membrane center, which determines the partitioning of O_2_ in the membrane. This led to the proposal of TC3 as the bead to represent molecular oxygen.

To further evaluate the TC3 bead, we computed the diffusion coefficient in water and hexadecane, the permeability through POPC and POPC:CHOL membranes, and the affinity for hydrophobic cavities of T4 lysozyme L99A. First, the diffusivity of the TC3 bead in both water and hexadecane was in fair agreement with those obtained from AA CHARMM simulations, reproducing the trend of a higher diffusivity in water than in hexadecane. A notable disparity occurred between the simulated results and experimental data, which is consistent with the tendency of simulations to excel in thermodynamic properties rather than kinetic ones.

Second, the permeability of the TC3 bead through the POPC and POPC:CHOL membranes was significantly higher than for the AA CHARMM and AA Amber force fields, potentially owing to reduced free energy barriers in the membrane headgroup in CG simulations. The discrepancy may also stem from the enhanced kinetics inherent to the CG resolution. Interestingly, experimental values showed a notably high *P* value, aligning closely with the CG result. However, one should keep in mind that comparing our results with experimental values may not be accurate due to the significantly smaller dividing surface in the EPR method compared to the membrane thickness. Third, to assess the accuracy of our model in protein simulations, we also examined the O_2_ model (TC3) binding to T4 lysozyme L99A. Our CG model demonstrated concordance with AA simulations of the work by Maeno et al., displaying enhanced O_2_ affinity for binding to the protein’s hydrophobic cavities compared to hydrophilic cavities.

The simulation of large biological systems can be challenging and even impossible to achieve with high-resolution AA MD because of the computational burden in terms of the memory load and computing time. The new CG model for O_2_ is compatible with the existing Martini 3 force field, and it will enable us to simulate large systems at reduced computational effort. While CG has a reduced spatial resolution, CG simulations will allow for the simulation of e.g., caveolae, where investigating O_2_ dynamics may help us understand its potential in the treatment of diseases such as caveolinopathies and pulmonary hypertension.^76,77^ Reassuringly, based on the current work, the TC3 bead was already used to study O_2_ partitioning into biomolecular condensates, showing a good match with experimental data.^78^

## Computational Details

The selection and validation of the CG bead for O_2_ are based on comparing experimental data, AA simulation data, and CG simulation data. Part of the simulations have been reported elsewhere, e.g., the CG simulations of the Martini 3 force field, which were complemented by several new simulations. This section reviews the simulation details of the new simulations.

### AA O_2_ Permeation through Lipid Membrane

Table 5 presents a detailed description of two AA-simulated systems, each composed of 1-palmitoyl-2-oleoyl-sn-glycero-3-phosphocholine (POPC) and cholesterol (CHOL) lipids. One box contains a homogeneous POPC membrane, while the other box contains a heterogeneous POPC:CHOL membrane. The systems included 0.15 M sodium chloride.

**Table 5:** Detailed Description of the Systems^a^

permeant	POPC	CHOL	regular CG W	tiny CG W	HD	Na^+^/Cl^–^	# permeants	*T* (K)	Δ*G*_*m*_ (*k*_*B*_*T*)	T4 lysozyme L99A
Free Energy and Permeability										
CG TC2	128		1395			14/14	18	310	4.06	
CG TC3	128		1395			14/14	18	310	3.22	
	96	32	1395			14/14	18	310	3.24	
CG TX1	128		1395			14/14	18	310	3.29	
CG TX2	128		1395			14/14	18	310	2.11	
AA CHARMM O_2_	128		5580			14/14	18	310	2.95	
	96	32	5580			14/14	18	310	3.05	
Diffusion										
CG TC3			68,400				10	310		
CG TC3					18,256		10	310		
Binding to Protein Cavities										
CG TC3			3250	1000		0/8	1	310		1

a “HD”, “W”, and “CHOL” refer to hexadecane, water, and cholesterol, respectively. Δ*G_m_* is the free energy difference for O_2_ between the center of the membrane (*z* = 0) and the water phase; in practice, it is computed from *F*(*z*) = −*k*_*B*_*T  *ln(*hist*(*z*)).

Utilizing Gromacs-2021.4,^79^ both systems underwent extensive AA MD simulations at 310 K. The simulations were conducted with the stochastic velocity rescaling thermostat with a 1.0 ps coupling constant.^80^ Periodic boundary conditions were applied in all three dimensions. To maintain pressure, the Parrinello–Rahman barostat was used, semi-isotropic pressure coupling was implemented with a reference pressure of 1 bar, a coupling constant of 5 ps, and an isothermal compressibility of 4.5 × 10^–5^ bar^–1^. Coulombic interactions were computed by using the particle mesh Ewald approach. At a 1.2 nm cutoff, both Coulombic and van der Waals potentials were smoothly shifted to zero by using force-switch modifiers at 1.0 nm. The lipid molecules were modeled using the CHARMM36 force field,^12^ and the oxygen molecules were modeled as a neutral molecule without quadropolar moment.^13^ Water was represented by the TIP3P model.^81^ The Verlet neighbor list algorithm with a cutoff of 1.2 nm was used. Integrating the equation of motion involved a time step of 2 fs with the leapfrog integrator. The center of mass motion was removed at each time step. Following the addition of oxygen molecules at a concentration of 86 μM, an energy minimization was executed. This concentration is selected to prevent the aggregation of O_2_ model beads.^13^ Subsequently, a 100 ns equilibration and a 1000 ns production run were conducted under constant pressure and temperature conditions (NPT ensemble). Snapshots of coordinates were saved at 1 ps intervals.

### New CG Simulations

A series of additional MD simulations with CG was performed to further validate the Martini 3 beads as models for molecular oxygen. In these simulations, the Martini 3 force field was again used.^41,82^ The systems were simulated using Gromacs-2021.4.^79^ The reaction-field approach was used to calculate the Coulombic interactions.^83^ With the potential-shift-Verlet modifiers, the van der Waals potentials were shifted to zero at a cutoff of 1.1 nm. The neighbor list was updated using the Verlet neighbor search algorithm with a cutoff length of 1.1 nm. The equation of motion was integrated using the leapfrog integrator with a time step of 20 fs. A temperature of 310 K and a pressure of 1 bar were set for all of the systems. The stochastic velocity rescaling thermostat was used with a coupling constant 1.0 ps.^80^ The Parrinello–Rahman barostat was used with a coupling constant of 12 ps. For systems with a membrane, semi-isotropic isothermal compressibility of 3 × 10^–4^ bar^–1^ was used. At every 100 time steps, the center of mass motion of the system was removed, and periodic boundary conditions were applied in all directions (*x*, *y*, *z*).

The next subsections specify the simulation boxes in the different types of simulations, as summarized in Table 5.

#### CG O_2_ Density

Two hundred Martini 3 O_2_ were placed in a 15 × 15 × 15 nm^3^ cubic box and subjected to energy minimization. Since the system contained gas, equilibration was performed in the NPT ensemble to allow for expansion. The box expanded to 21 × 21 × 21 nm^3^ and remained at this volume. To avoid large volume oscillations, equilibration was carried out in five steps, each with a 30 ns simulation time. During this process, the isotropic isothermal compressibility was gradually adjusted from an initial value of 2 × 10^–5^ bar^–1^ to a final value of 4.5 × 10^–5^ bar^–1^, and the barostat coupling constant was set to 50 ps to ensure constant pressure. Due to the presence of the gas phase, the neighbor list was updated at each simulation step. Following the equilibration, a 50 ns production run was conducted. The density was calculated using the “gmx energy” command of Gromacs.

#### CG O_2_ Permeation through the Lipid Membrane

Using CHARMM-GUI, the initial structures of the membranes were prepared.^84−87^ The exact same box compositions were used as in the AA membrane simulations of Section 5.1 for homogeneous POPC and heterogeneous POPC:CHOL membrane. As described in the Martini 3 documentation, the lipid models were obtained from the official repository.^88^ The Martini 3 model for cholesterol was used.^89^ As one CG water bead represents 4 water molecules, the CG simulation box contained 1395 CG water beads, whereas the AA simulation boxes contained 5580 water molecules (Table 5).

Systems were first energy minimized and then equilibrated in the NPT ensemble. The production run was 1000 ns of NPT simulation, and coordinates were stored every 1 ps for analysis.

#### CG O_2_ Diffusion in the Solvent

We conducted simulations with the CG models for O_2_ in a water box measuring 20.3 × 20.3 × 20.3 nm^3^ (∼68,400 water beads or ∼273,600 water molecules). The experimental solubility of O_2_ in water at 310 K and 1 bar is 1.3 × 10^–3^ mol/lit·atm as reported by Battino et al.,^49^ indicating that approximately 42,000 water molecules can dissolve a single O_2_ molecule. Our simulation box accommodated 10 CG beads for O_2_; although this brings the CG O_2_ concentration above the experimental O_2_ solubility, no clustering of these beads was observed, and this approach was maintained to improve the statistics. The same simulation is done with the CG O_2_ models immersed in a hexadecane box measuring 20.5 × 20.5 × 20.5 nm^3^ (∼18,256 hexadecane molecules) using the same computational settings.

For both systems, energy minimization, a 50 ns equilibration run, and a subsequent 200 ns production run were performed. The diffusion coefficient was determined by analyzing the mean square displacement (MSD) of O_2_ versus time plot. The diffusion coefficient is obtained from the slope of the linear regression applied to the MSD(t) plot from 1 to 50 ns. Using eq 14 from ref (90), the effect of periodic boundary conditions was accounted for in the CG systems, and the corrected values are presented in Table 2. The permeant’s radius was set to R = 1.7 nm, with box lengths (*L*) of 20.3 nm for the water box and 20.5 nm for the hexadecane box. For the AA systems in Table 2, the periodic boundary correction was also applied, with the same oxygen radius *R* and with the box length *L* was set to 5.0 nm for both the water and hexadecane box.^13^ Due to the hydrogen binding in water, the “stick” boundary condition was applied to calculate *D* in water, whereas the “slip” boundary condition was used for the HD system.

#### O_2_ Binding to Hydrophobic Cavities of T4 Lysozyme L99A

Two CG TC3 beads as O_2_ molecules were positioned within a dodecahedron box, containing one T4 lysozyme L99A protein, 3250 regular water Martini beads (13000 water molecules), 1000 tiny water Martini beads (2000 water molecules), and 8 chlorine ions to neutralize the system. Regular CG water beads, consisting of 4 water molecules, might be too bulky to adequately fill the hydrophilic cavities of the T4 lysozyme L99A protein. This could result in inaccuracies when simulating the binding of the O_2_ molecules to these protein cavities. To avoid this, 1000 tiny water beads were introduced to the system to compete with O_2_ for occupancy of the hydrophilic cavities; 29 such systems were created. Following a steepest-descent energy minimization run, each system underwent 200 ns of equilibration and a 2 μs NPT production run was performed. Frames were stored every 20 ps.

**Figure 4:**
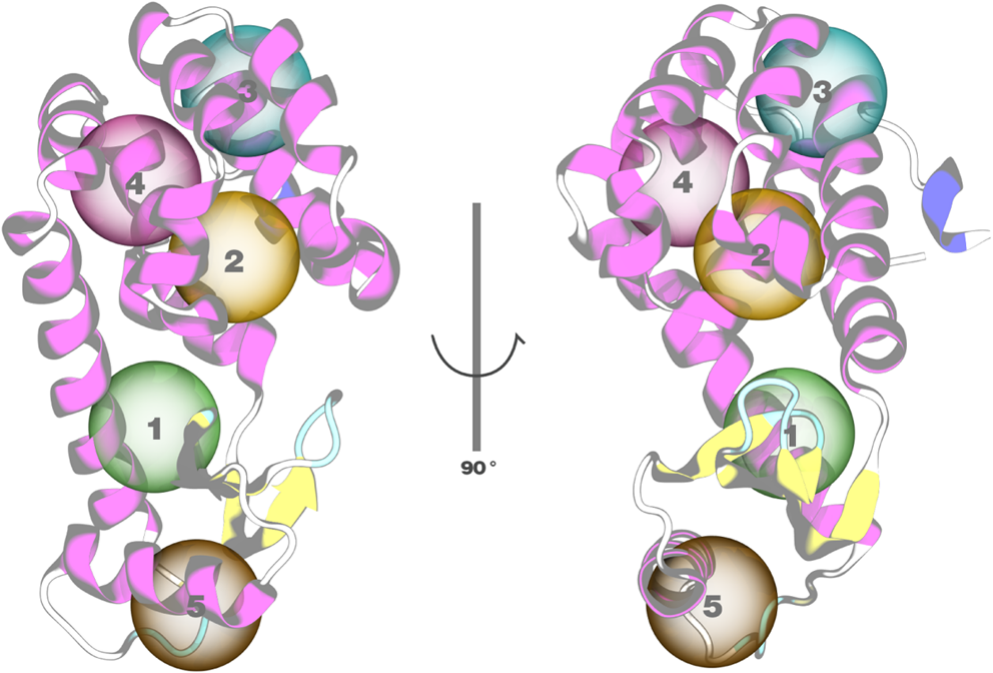
Locations of the five defined cavities: hydrophilic (1, 2), hydrophobic (3, 4), and random (5). Cavity 1: 28GLY, 29ILE, 63ALA, and 70ASP. Cavity 2: 101ASN, 102MET, 105GLN, 138TRP, and 149VAL. Cavity 3: 126TRP, 130ALA, 153PHE, and 154ARG. Cavity 4: 84LEU, 87VAL, 88TYR, 91LEU, 99ALA, 102MET, 111VAL, and 118LEU. Cavity 5: 50ILE, 51GLY, 52ARG, 53ASN, 54THR, 55ASN, 56GLY, 57VAL, 58ILE, and 59THR.

A concatenated, protein-centered trajectory of all the 29 systems was visualized in VMD, and an isosurface of O_2_ density was calculated using the “VolMap” extension of VMD.^91−93^ For each snapshot in each trajectory, the distance between oxygens and the centers of geometry of the five cavities (Figure 4) was computed using MDAnalysis.^94^ The residence time of O_2_ in each cavity was then estimated using a cutoff value of 6 Å. If any two registered binding events occurred within 60 ps of one another, they were considered to be part of the same binding event, and the residence times were computed as one event instead of two individual events. The ratio of the total time spent by oxygen in the cavities and the number of visits is used as an estimate of the average residence time of O_2_ in the cavity.

The free energy of O_2_ binding to each cavity was calculated using the following equation
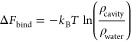
3where ρ_cavity_ is the density of O_2_ in the cavity, and ρ_water_ is the density of O_2_ in the water phase. The density of O_2_ in the water phase was calculated to be 0.283 kg/m^3^, while the density of O_2_ in each cavity was determined based on the number of snapshots where O_2_ is bound to the cavity and the volume of the sphere with radius 6 Å.
